# Hard X-ray nanofocusing at low-emittance synchrotron radiation sources

**DOI:** 10.1107/S1600577514016269

**Published:** 2014-08-29

**Authors:** Christian G. Schroer, Gerald Falkenberg

**Affiliations:** a Institut für Strukturphysik, Technische Universität Dresden, 01062 Dresden, Germany; b Deutsches Elektronen-Synchrotron DESY, Notkestrasse 85, 22607 Hamburg, Germany; c Fachbereich Physik, Universität Hamburg, Luruper Chaussee 149, 22761 Hamburg, Germany

**Keywords:** X-ray nanofocus, X-ray optics, diffraction-limited storage ring

## Abstract

X-ray scanning microscopy relies on intensive nanobeams generated by imaging a highly brilliant synchrotron radiation source onto the sample with a nanofocusing X-ray optic. Here, using a Gaussian model for the central cone of an undulator source, the nanobeam generated by refractive X-ray lenses is modeled in terms of size, flux and coherence. The beam properties are expressed in terms of the emittances of the storage ring and the lateral sizes of the electron beam. Optimal source parameters are calculated to obtain efficient and diffraction-limited nanofocusing. With decreasing emittance, the usable fraction of the beam for diffraction-limited nanofocusing experiments can be increased by more than two orders of magnitude compared with modern storage ring sources. For a diffraction-limited storage ring, nearly the whole beam can be focused, making these sources highly attractive for X-ray scanning microscopy.

## Abstract 2

X-ray scanning microscopy greatly benefits from a reduced emittance of synchrotron radiation sources, especially from a diffraction-limited storage ring. Nanofocusing is discussed in view of focus size, flux and coherence.

## 1. Introduction   

Hard X-ray scanning microscopy enjoys an increasing demand in many fields of science, such as physics and chemistry, biology, materials, earth and environmental science, and nanotechnology (Xu *et al.*, 2013 ▶: Vogt & Lanzirotti, 2013 ▶). Its key strength lies in the large penetration depth of hard X-rays that can penetrate specialized sample environments, such as chemical reactors, microfluidic or pressure cells. X-ray microscopy is thus ideally suited for *in situ* and *in operando* studies of physical and chemical processes. By using various X-ray analytical techniques as contrast, such as X-ray fluorescence, absorption or diffraction, X-ray scanning microscopy allows one to measure quantitatively the elemental composition, the chemical state or the local mesoscopic or atomic structure, respectively. In combination with tomography the three-dimensional inner structure of a specimen can be reconstructed.

In scanning microscopy, the signal-to-noise ratio and the minimal exposure time are limited by the flux in the probe beam. Therefore, it requires an intensive X-ray nanobeam. In order to achieve highest spatial resolutions, the X-rays from the source are imaged onto the sample in a diffraction-limited geometry. The beam size is then approximately given by the size of the Airy disc and the flux density mainly depends on the brilliance of the source and the transmission and numerical aperture of the optic. Diffraction-limited focusing is realised by matching the aperture of the nanofocusing optic to the lateral coherence length of the X-rays falling onto its aperture. This implies that only the coherent fraction of the X-ray beam can be efficiently focused. For modern synchrotron radiation sources, the coherent fraction of the beam lies in the range of per mille for hard X-rays (λ ≃ 1 Å) and thus the largest fraction of the beam cannot be used for nanofocusing. Modern synchrotron radiation sources are thus highly inefficient for X-ray scanning microscopy, yet still by far the best sources available today.

The important figure-of-merit of the source is the spectral brilliance, which describes the X-ray flux that is emitted by the source per phase space volume, *i.e.* per source size, solid angle and energy bandwidth. At fixed power of the source, the optimal brilliance is obtained when the source size and solid angle of emission are minimal. This is the case when the source is diffraction-limited, *i.e.* the source size and divergence match the intrinsic divergence of undulator radiation (*cf.* §2.1). Today’s synchrotron radiation sources have very different beam properties in the vertical and horizontal direction. While in the vertical direction the diffraction limit is reached in many cases even for hard X-rays, the horizontal source size and divergence is much larger than the diffraction limit. The larger horizontal emittance (*cf.* §2.1) can in principle be reduced by optimizing the electron optics in the storage ring, making the electron beam less divergent and confining it laterally to a smaller area. The X-ray microscopist’s dream would be a diffraction-limited storage ring (DLSR), where also the horizontal beam size and divergence would match the intrinsic divergence of the undulator radiation.

In this article we investigate the nanobeam properties, such as beam size, flux and coherence, in terms of the source parameters. This can be done analytically within a Gaussian model. For a given undulator source, the optimal storage ring parameters are calculated to optimize the source for scanning microscopy. We show that a reduction in source emittance significantly improves the efficiency of nanofocusing and that ultimately, for a DLSR, the nanofocusing efficiency could be increased by more than two orders of magnitude compared with what is possible today.

## 2. X-ray scanning microscope   

**Figure 1:**
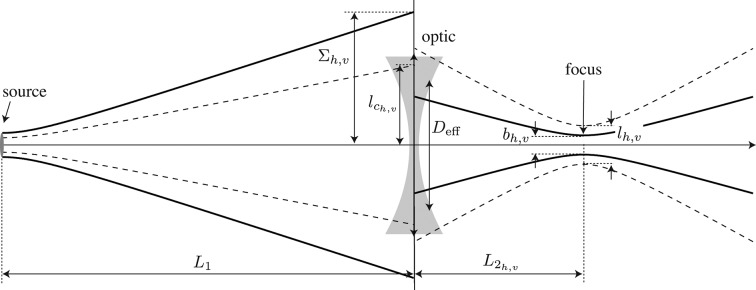
Focusing hard X-rays from a partially coherent synchrotron radiation source into a nanobeam. 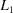 and 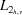 are the source-to-optic and optic to-focus distance, respectively. 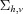 is the r.m.s. beam size and 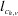 the lateral coherence length before the nanofocusing optic in the horizontal and vertical direction, respectively. 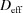 is the effective aperture of the nanofocusing optic, and 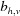 and 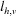 are the horizontal and vertical (FWHM) beam size and lateral coherence length in the nanofocus, respectively.

X-ray scanning microscopes rely on a laterally small probe beam that is generated by imaging the source onto the sample in a strongly demagnifying geometry. The nanobeam properties are determined by the relation of the effective aperture of the nanofocusing optic to the beam size, wavefront curvature and lateral coherence length at its entrance. In many cases, scanning microscopes make use of a secondary source to optimally match the above-mentioned quantities to each other. Here, for the simplicity of the presentation, we will consider a simpler imaging scheme, imaging the source directly onto the sample by one nanofocusing optic (*cf.* Fig. 1 ▶).

We model monochromatic X-rays as a scalar wavefield that propagates according to the Helmholtz equation (Born & Wolf, 1999 ▶). This assumes that polarization effects can be neglected. In a straight focusing geometry (Fig. 1 ▶) and considering maximal deflection angles in the range of several milliradians, the paraxial approximation is well justified and the polarization can indeed be neglected. In the following, we model the radiation in the central cone of an undulator source by a Gaussian ensemble of Gaussian limited waves (§2.1). We then propagate this Gaussian ensemble to the refractive lens (§2.2), model the lens in terms of a thin object (§2.3) and propagate the X-rays to an arbitrary distance behind the lens, in particular into the focal plane (§2.4). Minimal focus sizes are obtained in a so-called diffraction-limited imaging geometry that is discussed in §2.5.

### 2.1. Gaussian model for an undulator source   

In the undulator of a synchrotron radiation source the electrons radiate independently of each other and in an uncorrelated fashion. The radiation emitted by an electron is strongly directed into the forward direction by the relativistic aberration and interference of the emission amplitudes for the different poles of the undulator. The resulting root-mean-square (r.m.s.) opening angle of the emission cone is (Thompson *et al.*, 2009 ▶) 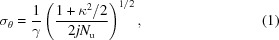
where γ is the electron energy relative to its rest mass, κ is the undulator parameter, *j* is the integer number describing the harmonic of the radiation, and 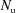 is the number of undulator periods. The most important parameters and quantities considered in this article are listed for quick reference in Table 1 ▶. The spectrum of the undulator radiation on the optical axis is concentrated in odd harmonics,
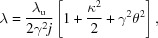
with λ being the wavelength of the X-rays, 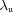 the undulator period, and θ the emission angle relative to the undulator axis.

**Figure 2:**
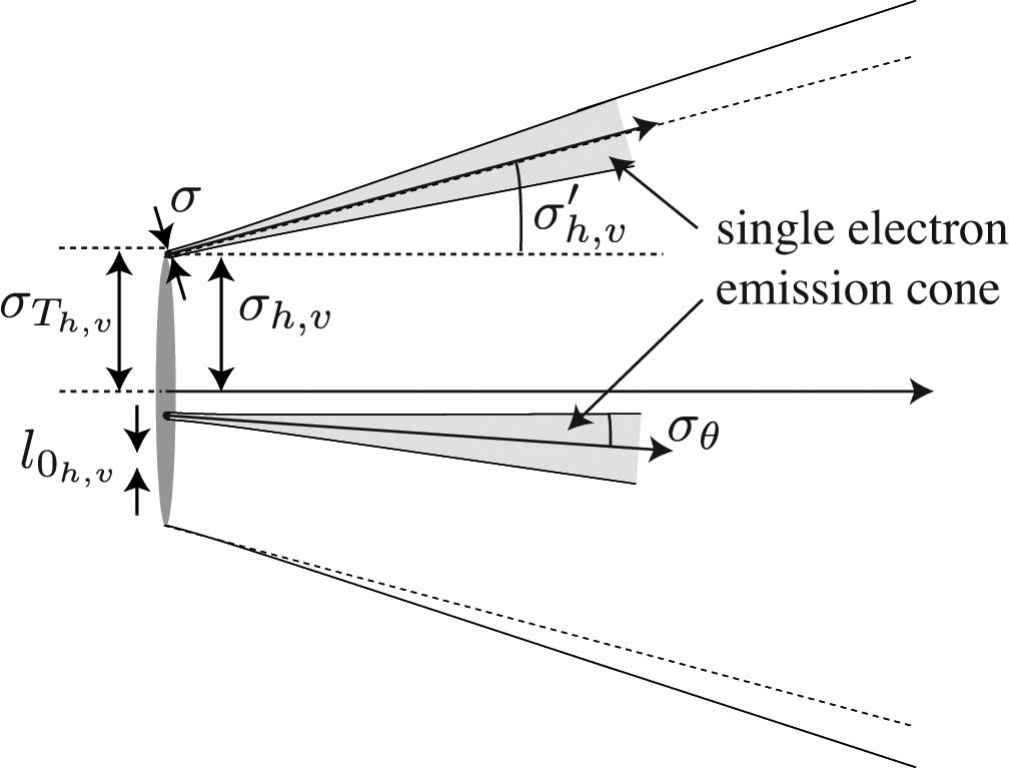
Schematic sketch of the undulator source model with its parameters. 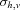 is the r.m.s. lateral size of the electron beam, σ the r.m.s. diffraction-limited source size, and 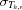 the overall r.m.s. lateral size of the source. 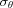 is the natural divergence of the undulator radiation and 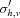 the r.m.s. divergence of the electron beam. 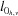 is the lateral coherence length of the source.

To model the emission of a single electron at a transverse position 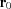 and moving into a certain direction relative to the optical axis, the electromagnetic wavefield of the X-rays in the undulator can be described by a complex scalar amplitude, 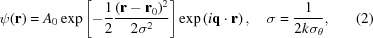
where 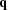 is the transverse wavenumber defining the propagation direction of the electron in terms of photon momentum components 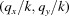 with 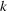 = 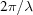. 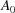 is the amplitude and σ the r.m.s. diffraction-limited source size for the synchrotron radiation emitted by one electron. The Gaussian model for undulator radiation is only valid in the far-field of the undulator, *i.e.* at distances that are much larger than the length of the undulator. Two exemplary electrons and their emission cones are shown in Fig. 2 ▶.

In the storage ring the electrons are confined into bunches, filling a certain region of phase space. In the transverse direction the distribution of electrons can be modeled by a Gaussian ensemble that is defined by the electron density in lateral space and momentum, given by 
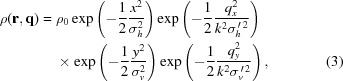
where 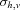 and 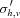 are the r.m.s. source size and divergence in the horizontal and vertical direction, respectively (*cf.* Fig. 2 ▶). If the dynamics of the electrons in the storage ring decouple in the horizontal and vertical direction, the respective emittances 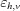 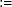 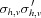 are constants of motion around the storage ring.

For the purposes of this article, we can describe the radiation from the ensemble of electrons by the mutual intensity function (Born & Wolf, 1999 ▶) 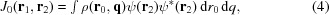
that describes the time-averaged correlation between the field amplitudes at the transverse positions 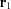 and 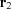.

Due to the symmetry of the Gaussian model and the source the mutual intensity function can be separated into the product of two functions that describe the horizontal and vertical beam properties, respectively, 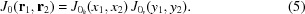
Inserting (2) and (3) into (4), we obtain 
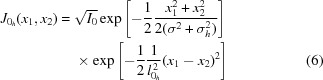
and an analogous expression for 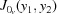. 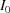 is the maximal intensity in the source plane. Here, 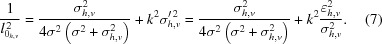
Equation (6) can be interpreted in the following way: for 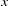 = 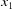 = 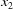 the mutual intensity yields the intensity of the wavefield at the location *x*. The convolution of the Gaussian emission cone with the Gaussian electron density distribution yields an r.m.s. source size of (*cf.* Fig. 2 ▶) 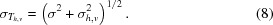
The second exponential term in (6) describes an exponential decay of the amplitude–amplitude correlation with increasing distance 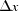 = 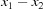 in the source. 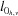 is the characteristic correlation length and is called the lateral coherence length in the given direction. It is a function of both the source size and the electron beam divergence.

The source is considered diffraction-limited when 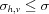 and 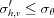. Making use of the relation between σ and 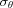 in (2) a diffraction-limited emittance fulfills the following condition (Thompson *et al.*, 2009 ▶),
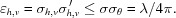
In the diffraction-limited case [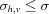, 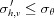 in (7) and (8)], the lateral coherence length 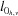 in the source exceeds the source size 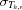 by at least 15%. For current synchrotron radiation sources, however, 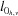 is much smaller than the source size (*cf.* Fig. 2 ▶).

### 2.2. Propagation of the X-rays to the nanofocusing optic   

Equation (6) has the Gaussian structure of a so-called Gaussian shell model (Vartanyants & Singer, 2010 ▶; Singer & Vartanyants, 2014 ▶). This model has been analyzed in detail by I. Vartanyants and A. Singer, including the focusing of a partially coherent beam by refractive X-ray lenses (Singer & Vartanyants, 2014 ▶). Here, we calculate the mutual intensity function in analogy to Singer & Vartanyants (2014 ▶), expressing the results in terms of the source parameters described in the previous section. It is useful to follow the propagation of the beam once again to identify the important parameters and their dependence on the source.

In a first step, the mutual intensity in the source plane given by (6) is propagated to the entrance of the focusing optic located at a distance 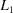 from the source (Born & Wolf, 1999 ▶),
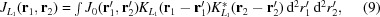
where 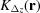 is the Fresnel propagator of the X-rays along the optical axis. In the paraxial Fresnel–Kirchhoff approximation it is given by
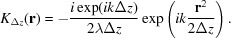
The integral (9) can be separated into a product of a horizontal and vertical contribution that, again, can be treated separately in the following.

The horizontal contribution is 
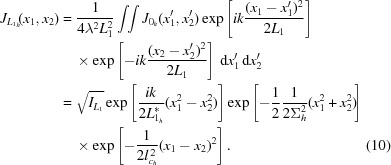
A similar expression is found for the vertical direction. As opposed to the wavefield in the source plane, the mutual intensity at a distance 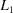 from the source includes an additional phase term that describes the wavefront curvature. The wavefront is curved with a curvature radius that describes the effective source distance,
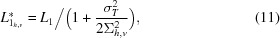
that is slightly shorter than the geometric distance 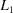 from the source to the optic and can be slightly different for the horizontal and vertical direction, introducing a slight astigmatism in that case. For current sources, however, the effect is so small that it is not easily observable and can be neglected.

The r.m.s. beam size for the intensity just before the nanofocusing optic (Fig. 1 ▶) is 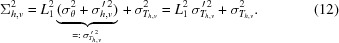
It can be easily interpreted as the width of the convolution of the single electron emission cone with the spatial and angular distribution of the source ensemble defined by equation (3). The products 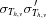 are called the transverse emittances of the photon beam. The brilliance of the source is inversely proportional to both these transverse emittances (Thompson *et al.*, 2009 ▶). The lateral coherence length is 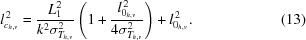
While 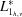 determines the position of the focal spot along the optical axis, 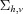 and 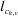 determine the transmission through the optic and the beam size in the focus. These three quantities fully determine the focal properties. 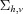 and 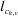 are shown in Fig. 1 ▶.

### 2.3. Focusing by parabolic refractive X-ray lenses   

There are many different nanofocusing X-ray optics available today, exploiting reflection (Jarre *et al.*, 2005 ▶; Mimura *et al.*, 2007 ▶), diffraction (Chu *et al.*, 2008 ▶; Kang *et al.*, 2008 ▶; Mimura *et al.*, 2010 ▶; Vila-Comamala *et al.*, 2011 ▶; Yan *et al.*, 2011 ▶) and refraction (Schroer *et al.*, 2005 ▶, 2011 ▶, 2013 ▶). Nanofocusing refractive X-ray lenses are used in the scanning microscopes at beamline ID13 of the ESRF, and beamline P06 of PETRA III (Schroer *et al.*, 2010 ▶). For the purpose of this article, it is useful to consider these refractive X-ray optics, as their aperture function is intrinsically Gaussian.

Parabolic refractive X-ray lenses and their imaging properties were previously described in detail (Lengeler *et al.*, 1999 ▶; Kohn, 2003 ▶; Schroer *et al.*, 2013 ▶). In the case of nanofocusing, they can typically not be considered as thin optics. However, their particular imaging properties (Kohn, 2003 ▶; Schroer *et al.*, 2013 ▶) allow one to replace the thick optic with an effective Gaussian thin-object transmission model in the case of nanofocusing, *i.e.* when the source-to-lens distance 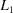 is much larger than the focal length.

In general, the aperture of the nanofocusing optic acts like a spatial filter, truncating part of the electromagnetic wave in the plane of the optic. For a thin refractive focusing optic with focal length *f*, the transmission function is given by 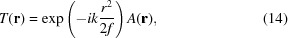
where 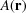 is a (potentially complex) aperture function of the optic. For parabolic refractive X-ray lenses that are free of aberrations, 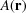 is real and given by 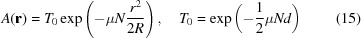
inside the geometric aperture radius 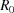 and zero outside (Lengeler *et al.*, 1999 ▶). 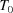 is the transmission of the X-ray lenses on the optical axis and can be minimized by reducing the distance *d* between the apices of the parabolae (Lengeler *et al.*, 1999 ▶). The properties of the lens material enter in equations (14) and (15) implicitly *via* *f* and μ.

Here, we consider the case where 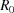 is sufficiently large to justify a fully Gaussian aperture [*cf.* Schroer *et al.* (2013 ▶) for examples of nearly Gaussian nanobeams generated by refractive nanofocusing lenses]. In this case, the aperture is limited by Gaussian absorption inside the lens material. *N* is the number of single lenses and *R* the radius of curvature of an individual lens surface (Lengeler *et al.*, 1999 ▶). The effective aperture is defined as (Lengeler *et al.*, 1999 ▶) 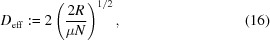
and describes the 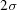 value of the width of the transmission of the amplitudes (not intensities) through the lens (Lengeler *et al.*, 1999 ▶) and is thus 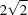 times larger than the r.m.s. width of the intensity transmission profile of the lens.

### 2.4. Propagating the X-rays through the caustic of the nanobeam   

The mutual intensity at the distance 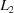 after the lens is given by 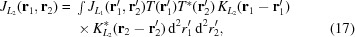
and by symmetry of the problem can again be separated into a horizontal and vertical contribution [*cf*. (5)]. An arbitrary distance 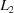 behind the optic the horizontal part of the mutual intensity is 
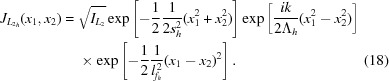
The vertical part 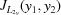 has the same structure. The first exponential term in (18) describes the lateral beam size. The r.m.s. beam size is
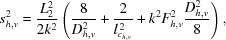
where 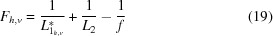
describes the defocus and 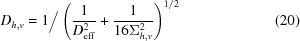
is the effective aperture corrected for the inhomogeneous Gaussian illumination with the r.m.s. width 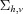.

The second exponential in (18) describes the wavefront curvature with a curvature radius
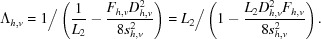
The last exponential term in (18) describes the average amplitude–amplitude correlation with a lateral coherence length
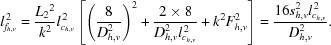
In the focus, 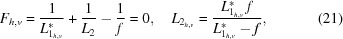
and the wavefront is flat, *i.e.* 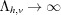. In the case that 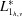 [*cf.* (11)] deviates significantly from the geometric distance 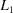, the focusing becomes intrinsically astigmatic and the horizontal and vertical focus do no longer coincide at a common position 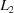 along the optical axis.

In the focus, the full width at half-maximum (FWHM) lateral beam size 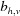 is minimal,
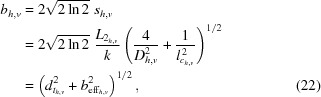
and has two contributions:

(i) The first contribution is the size of the Airy disc, 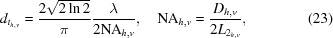
described by Abbe’s formula (Lengeler *et al.*, 1999 ▶). Here, 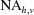 is the numerical aperture. The effective aperture 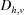 defined in (20) depends not only on the Gaussian aperture function (15) of the optic but is also affected by the Gaussian beam illuminating the optic. As long as the aperture of the optic is illuminated homogeneously, *i.e.* 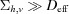, the effective aperture is determined by the attenuation in the lens material only. If, however, the beam size 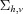 is comparable with the effective aperture 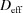 of the optic, the illumination of the aperture also influences the diffraction limit. In the other extreme case, *i.e.* for large aperture optics with 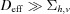, the diffraction limit is solely determined by 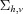.

(ii) The second contribution is the effective geometric beam size,
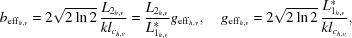
that is the image of an effective source with FWHM extension 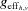 demagnified by the factor 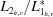. In the case of a perfectly incoherent source, 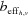 is the geometric image of the source.

**Figure 3:**
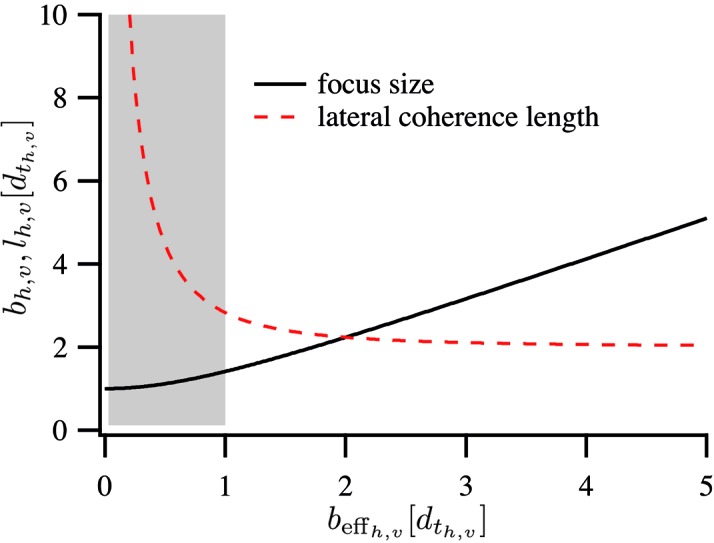
Focus size 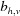 and lateral coherence length 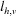 in the focus as a function of effective geometric image size 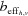. All quantities are in units of the size 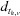 of the Airy disc. The gray shaded area is the regime of diffraction-limited focusing (*cf.* §2.5).

In the focal plane, the FWHM lateral coherence length reduces to 
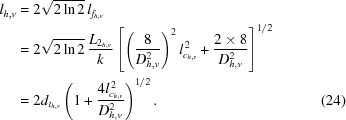
In units of the diffraction limit 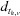, the beam size and the lateral coherence length have the form 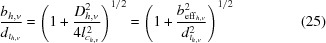
and 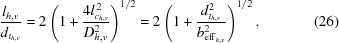
respectively. They depend only on the ratio of 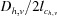 or equivalently 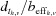. Fig. 3 ▶ shows this generic dependence of 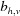 and 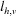 on the effective image size of the source.

**Figure 4:**
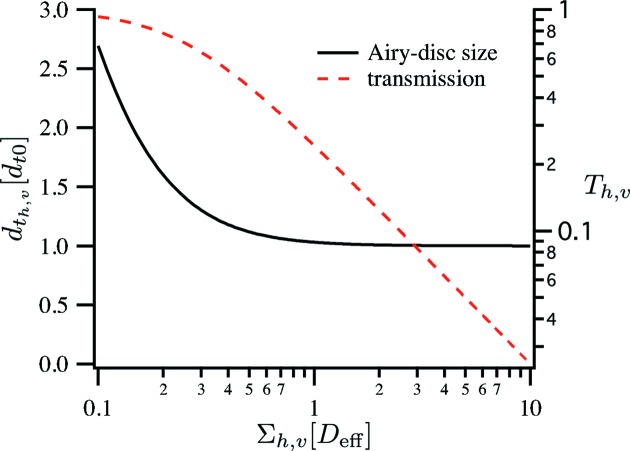
Dependence of the Airy-disc size 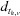 and the transmission 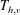 through the optic on the illumination 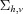 of the effective aperture 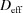 of the optic.

The Airy-disc size 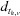 depends mainly on the effective aperture 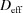 [equation (16)] and on the beam size 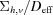 relative to the effective aperture,
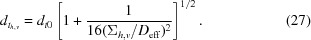
Here, 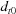 is the diffraction limit for homogeneous illumination of the aperture (Lengeler *et al.*, 1999 ▶). Fig. 4 ▶ shows this dependence expressed in (27). A significant increase of 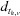 only occurs when the beam size 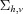 is significantly smaller than the aperture 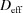 of the optic. In the more common case, where the aperture is fully illuminated, the diffraction limit is nearly independent of the illumination.

The efficiency of a focusing optic is usually defined by the ratio of the transmitted flux and that incident on the aperture of the optic. Here, we want to analyze what fraction of the undulator radiation can be focused by the optic. For this purpose, we consider the ratio of the *total* flux 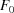 before and 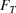 after the optic that can, again, each be separated into a horizontal and vertical contribution due to the symmetry of the Gaussian model. The horizontal contribution is
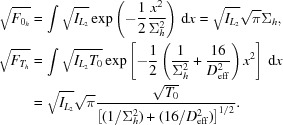
Analogous expressions are found for the vertical contribution. The transmission *T* separates into a product of two one-dimensional transmission functions, 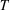 = 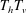, with 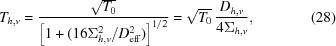
where 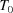 is given in equation (15). Just like the Airy-disc size, these two quantities depend on the ratio of 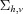 and 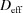 only. Fig. 4 ▶ shows the dependence of 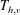 [given in (28)] on the illuminating beam size 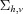. To simplify the presentation, we set the constant 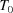 = 1 in Fig. 4 ▶ and in the rest of the article. This corresponds to the case in which the refractive lenses have a negligible thickness *d* on the optical axis.

The flux density in the focus determines the quality of the nanoprobe. From (18) follows
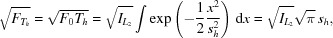
and a similar expression for 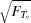. The maximal intensity in the focal plane is thus given by 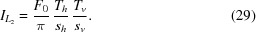


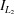
 is maximized by maximizing 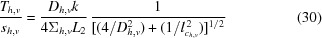
for both *h* and *v*. For given effective aperture 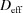 and emittance there is an optimal source size. For given source parameters, the optimum is reached for 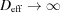, *i.e.* for an optic that can capture the full beam.

### 2.5. Diffraction-limited focusing   

As the effective geometric image size 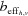 is made smaller and smaller, the beam size 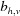 [*cf.* equation (22)] is eventually dominated by the size of the Airy disc 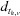. This can be expressed in the form 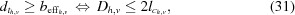
and serves here as a definition for diffraction-limited focusing. In this case, the transmission is 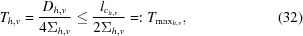
where 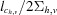 is the maximal fraction of the beam that can be focused at the diffraction limit.

In Fig. 3 ▶, diffraction-limited focusing is achieved in the gray shaded region. The smaller the effective image 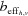 of the source, the higher becomes the degree of lateral coherence. This is important for coherent X-ray diffraction imaging. In these techniques, the sample is illuminated with coherent X-rays and a far-field diffraction pattern is recorded. There are several techniques, amongst which scanning coherent diffraction microscopy, also called ptychography, is one of the most successful (Thibault *et al.*, 2008 ▶; Schropp *et al.*, 2011 ▶, 2012 ▶; Dierolf *et al.*, 2010 ▶; Giewekemeyer *et al.*, 2010 ▶, 2011 ▶; Wilke *et al.*, 2012 ▶; Holler *et al.*, 2014 ▶).

The statistics of a signal in the diffraction pattern that comes from a certain feature in the sample depend on the fluence on this feature during the exposure of the diffraction pattern (Schropp & Schroer, 2010 ▶). This leads to a trade-off between maximizing intensity and lateral coherence. While there are algorithms that can cope with reduced coherence (Thibault & Menzel, 2013 ▶), so far the best results have been obtained with highly coherent beams, *i.e.* when the lateral coherence length is much larger than the focus size (Schropp *et al.*, 2010 ▶).

It is thus useful to introduce a more general criterion for diffraction-limited focusing, requiring 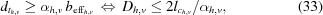
where 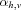 determines the degree of coherence. The condition 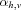 = 1 corresponds to the diffraction-limited focusing introduced in (31). For 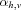 > 1, the coherence in the focus is increased, reducing the transmission through the optic,
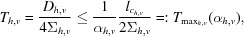
accordingly. For an increase in coherence length by 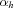 and 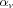 in the horizontal and vertical direction, respectively, the transmission is reduced by 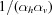.

## 3. Influence of emittance and source size on the properties of the nanobeam   

In the following the influence of the source on the nanobeam properties is discussed. The source properties are significantly different for the horizontal and vertical direction and are, therefore, treated separately. In the Gaussian model for a synchrotron radiation source presented in §2.1, the source is parameterized by the emittance 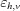 and the source size 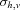. The diffraction limit of the source is determined by 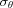 [*cf.* equation (1)].

The quantitive results in the following are calculated for a 6 GeV storage ring and a photon energy of 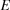 = 12.4 keV (wavelength λ = 1 Å). For this energy a spectroscopy undulator source at PETRA III has the diffraction-limited divergence of 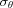 = 7.0 µrad on the third harmonic [calculated using equation (1) with parameters from Barthelmess *et al.* (2008 ▶)].

### 3.1. Horizontal focusing   

**Figure 5:**
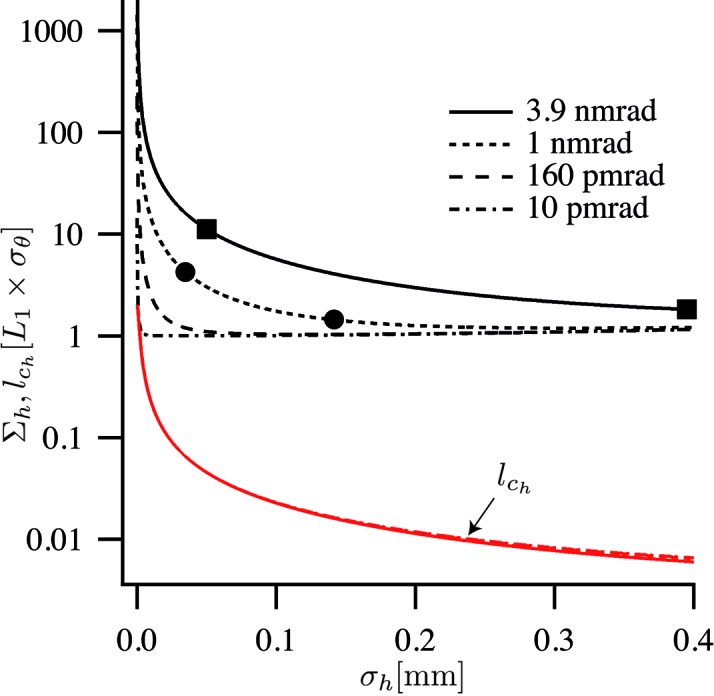
Horizontal beam size 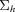 and lateral coherence length 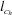 at a nanofocusing optic as a function of source size 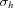 for four different emittances. The squares indicate parameter values comparable with those in the ESRF low- and high-β sections (Sette, 2012 ▶), the circles those in low- and high-β sections at PETRA III (http://photon-science.desy.de/facilities/petra_iii/machine/parameters/index_eng.html). The lateral coherence length 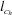 is in general much smaller than the beam size and varies only weakly with the emittance.

For nanofocusing, the beam size 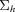 and lateral coherence length 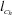 at the optic are the crucial parameters, compared with the effective aperture 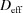. Fig. 5 ▶ shows the beam size 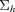 given by (12) as a function of source size 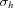 for four representative horizontal emittances. The 3.9 and 1 nm rad are horizontal emittances realised by ESRF and PETRA III, respectively. 160 pm rad is the target value for the horizontal emittance for the ESRF within the upgrade proposal for Phase II (Sette, 2012 ▶). The 10 pm rad corresponds to a nearly diffraction-limited source at a wavelength of λ = 1 Å (*E* = 12.4 keV).

The lateral coherence 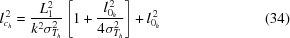
is largely dominated by the first term, as the lateral coherence length 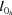 in the source is small, typically only a few hundred nanometers in size for hard X-rays. Its contribution is only relevant near the diffraction limit, when 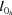
≃ 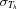 [second term in (34)], or for very large source sizes, when the third term in (34) becomes comparable with the first one. Fig. 5 ▶ shows 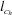 given in (34) for the four different emittances. The lateral coherence length is in most cases much smaller than the lateral beam size, except for the diffraction-limited case (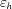 = 10 pm rad), where the coherence length reaches the beam size for small 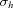.

**Figure 6:**
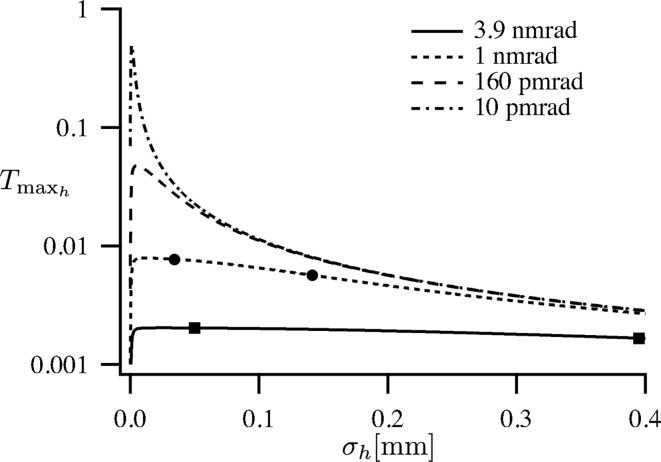
Fraction of the radiation emitted from the source and optimally focused to the diffraction limit in the horizontal direction. The squares and dots mark the parameters of the ESRF and PETRA III for the high- and low-β cases, respectively.

The fraction of the beam that can be focused to the diffraction limit is always smaller than 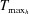 = 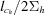 as given in (32). It is depicted in Fig. 6 ▶. The dependence of 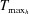 as a function of 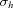 is relatively flat for the current sources and, therefore, efficient nanofocusing can be realised over a large range of beam sizes. The fraction of the beam that can be focused to the diffraction limit is nearly independent of the β function.

For sources with a smaller emittance, however, the optimum for the focused fraction 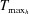 as a function of source size 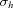 becomes more and more pronounced. In the 160 pm rad case, the optimal source size lies at 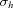 = 5.1 µm and would result in an improvement in efficiency by a factor of 6.1 compared with the PETRA III low-β case. In the diffraction-limited case (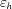 = 10 pm rad), optimal focusing would be reached for a source size of 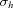 = 2.75 µm with 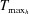 = 0.49. The focusing would be about a factor 64 times more efficient in the horizontal direction.

### 3.2. Vertical focusing   

**Figure 7:**
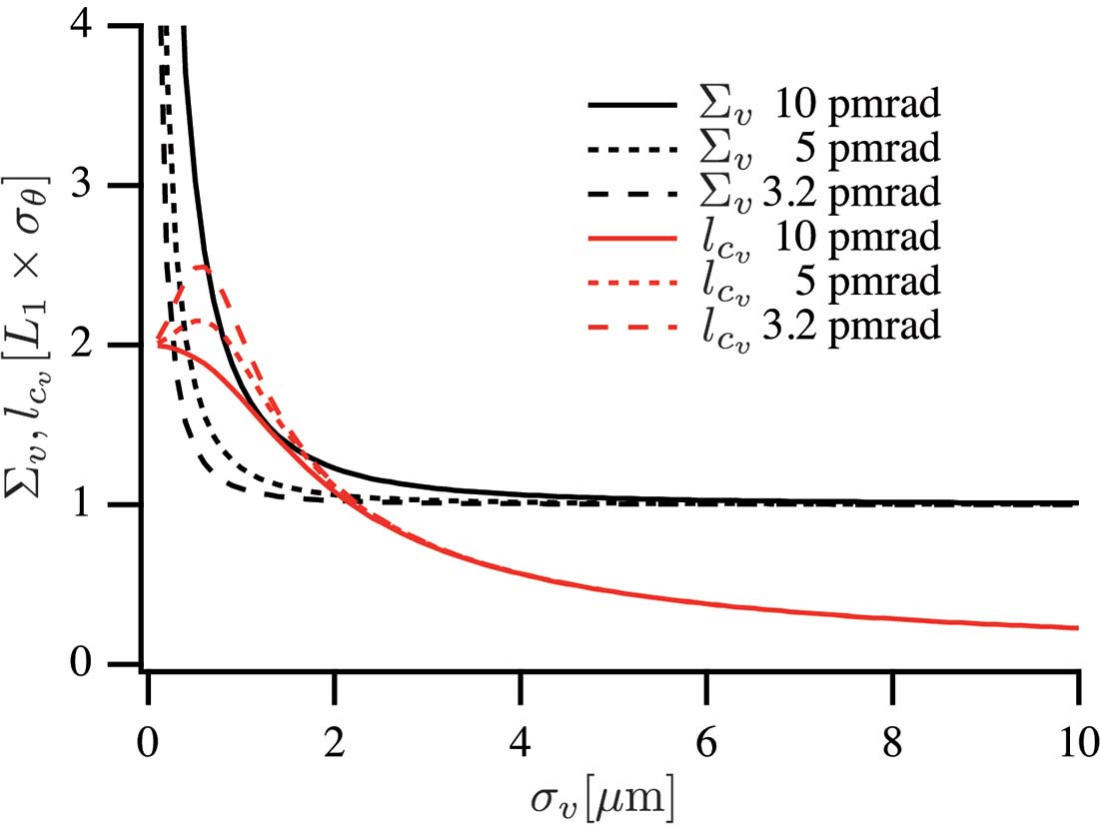
Vertical beam size 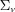 and lateral coherence length 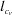 at a nanofocusing optic as a function of source size 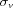 for three different emittances.

Currently, modern synchrotron radiation sources are not far from being diffraction-limited in the vertical direction at 12.4 keV. Fig. 7 ▶ shows the lateral beam size 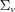 and coherence length 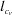 given by equations (12) and (13), respectively, as a function of source size for the vertical emittances 10 pm rad (PETRA III), 5 pm rad (minimal coupling at ESRF) and 3.2 pm rad (ESRF Upgrade II), respectively. For the 5 pm rad and 3.2 pm rad cases the lateral coherence length exceeds the beam size for certain source sizes 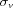. This illustrates the high degree of lateral coherence in the vertical direction.

**Figure 8:**
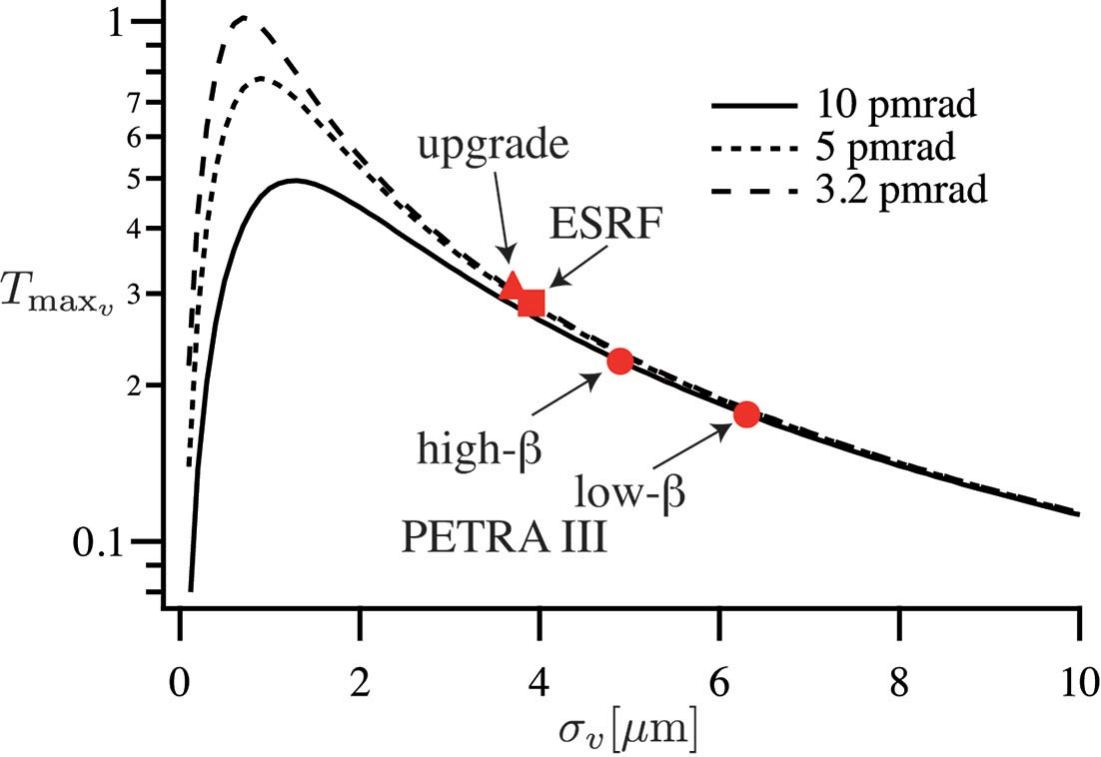
Fraction of the radiation emitted from the source focused to the diffraction limit in the vertical direction. The squares and dots mark the parameters of the ESRF and PETRA III for the high- and low-β cases, respectively, the triangle marks the parameters for the ESRF Upgrade II (Sette, 2012 ▶).

Fig. 8 ▶ shows the maximal transmitted fraction 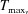 of the undulator beam [given by (32)] that can be focused to the diffraction limit. The dots and squares indicate the PETRA III and ESRF vertical source sizes in high- and low-β sections. In terms of optimal use of the beam, the currently given and targeted source sizes are slightly too large, not taking full advantage of the full coherence in the beam. As the lateral coherence 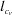 can exceed the beam size, large aperture optics that do not cut into the beam can be used in this case. Nearly the full beam can be focused to the diffraction limit in the vertical direction.

The full transmission is calculated as the product 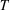 = 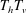. Table 2 ▶ summarizes the maximal fraction of the beam that can be focused to the diffraction limit for different existing and potential future synchrotron radiation sources.

## 4. Conclusion   

Today, diffraction-limited focusing is very inefficient. Due to the relatively large horizontal emittance of modern synchrotron radiation sources, only a few photons in a thousand that are emitted from the undulator can at best be transferred into the nanofocus. Future low-emittance storage rings can significantly increase the focusing efficiency. In the diffraction limit, nearly the whole central cone of the undulator could be efficiently focused to the diffraction limit, increasing the focusing efficiency by almost three orders of magnitude.

When the lateral coherence length 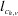 exceeds the beam size 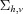 by a factor of two, *i.e.*

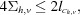
the full beam can be focused to the diffraction limit by a large-aperture optic, for which 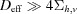 [*cf.* equation (31)]. According to Fig. 7 ▶, this condition can be fulfilled for sufficiently small emittances (in our numerical example for 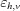 ≃ 5 pm rad or smaller) and an optimized source size. An example of such a large aperture optic could be a large Kirkpatrick–Baez multilayer mirror system (Mimura *et al.*, 2010 ▶) that barely truncates the Gaussian beam. For this optic, the diffraction-limiting aperture is defined by the Gaussian beam profile at the optic and 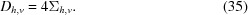
Inserting (35) into (23), the diffraction-limited beam size is
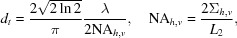
the ultimate intensity is independent of the aperture and given by 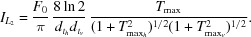
With 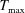 = 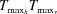 ≃ 1, it merely depends on 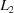 and thus on the focal length *f* of the optic.

For a numerical aperture of NA = 1 mrad and a photon flux of 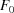 = 10^13^ photons s^−1^, a maximal intensity of 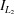 = 6.3 × 10^9^ photons s^−1^ nm^−2^ in a FWHM spot of 53 nm × 53 nm could be reached, exceeding current coherent nano­probe intensities by at least three orders of magnitude.

The increase in flux in the nanobeam would allow for fast scanning and higher signal-to-noise ratios, enabling fast imaging of dynamical processes with various X-ray analytical contrasts. For coherent X-ray diffraction imaging and ptychography, the sensitivity and spatial resolution could be improved to resolve features inside an object that are down to below 1 nm in size. These significant improvements for nanofocusing require an enormous effort to realise a truly diffraction-limited storage ring source. In addition, significant advances are also needed in the field of beamline optics and their stability. Important steps towards this ultimate goal are the construction of the new synchrotron radiation sources MAX IV in Lund, Sweden, and NSLS-II at Brookhaven National Laboratory, USA, and the future upgrades of the European Synchrotron Radiation Facility in Grenoble, France, and of the Advanced Photon Source at Argonne National Laboratory near Chicago, USA. With its large circumference of about 2.3 km, PETRA III at DESY in Hamburg, Germany, is well suited for a potential upgrade into a diffraction-limited storage ring. 

## Figures and Tables

**Table 1:** List of parameters and quantities

Quantity	Unit	Definition	Description
Storage ring and source			
γ	1	Equation (1)	Relativistic parameter: energy of electrons relative to their rest mass
κ	1	Equation (1)	Undulator parameter
*j*	1	Equation (1)	Integer number describing the harmonic of the undulator radiation
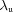	m	§2.1	Undulator period
θ	rad	§2.1	Angle measured relative to optical axis
*E*	eV		Energy of X-rays
λ	m	§2.1	Wavelength of X-rays
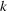 = 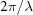	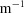	§2.1	Wavenumber of X-rays
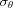	rad	Equation (1)	r.m.s. divergence of the single-electron emission cone (intensity)
σ	m	Equation (2)	r.m.s. diffraction-limited source size (intensity) as a result of limited divergence 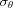
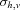	m	Equation (3)	Horizontal and vertical r.m.s. lateral size of the distribution of electrons in the undulator
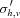	rad	Equation (3)	Horizontal and vertical r.m.s. lateral divergence of the distribution of electrons in the undulator
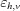	m rad	§2.1	Horizontal and vertical emittance
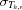	m	Equation (8)	Horizontal and vertical r.m.s. lateral source size in undulator
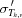	rad	Equation (12)	Horizontal and vertical r.m.s. beam divergence in undulator
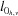	m	Equation (7)	Horizontal and vertical r.m.s. coherence length in the source
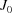	[intensity]	Equation (4)	Mutual intensity function in the source plane
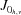	[intensity]^1/2^	Equation (5)	Horizontal and vertical factor of mutual intensity function in the source plane

Beam properties before the nanofocusing optic and properties of the nanofocusing optic			
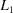	m	§2.2	Source-to-optic distance
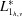	m	Equation (11)	Horizontal and vertical effective source-to-optic distance
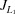	[intensity]	Equation (9)	Mutual intensity just before the nanofocusing optic
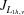	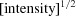	Equation (10)	Horizontal and vertical factor of mutual intensity just before the nanofocusing optic
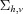	m	Equation (12)	Horizontal and vertical r.m.s. beam size (intensity) just before the nanofocusing optic
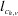	m	Equation (13)	Horizontal and vertical lateral coherence length just before the nanofocusing optic
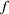	m	Equation (14)	Focal length of the nanofocusing optic
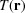	1	Equation (14)	Complex transmission function of the nanofocusing optic
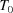	1	Equation (15)	Transmission of refractive lens on the optical axis
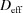	m	Equation (16)	Effective aperture of the refractive lens

Beam properties of caustic			
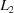	m	§2.4	Arbitrary distance behind the nanofocusing optic
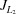	[intensity]	Equation (17)	Mutual intensity at distance 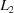 behind the nanofocusing optic
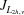	[intensity]^1/2^	Equation (18)	Horizontal and vertical factor of mutual intensity at distance 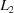 behind the nanofocusing optic
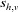	m	§2.4	Horizontal and vertical r.m.s. beam size at a distance 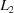 behind the nanofocusing optic
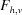	m^−1^	Equation (19)	Horizontal and vertical defocus at distance 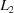 behind the nanofocusing optic
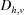	m	Equation (20)	Horizontal and vertical effective aperture corrected for Gaussian illumination
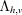	m	§2.4	Horizontal and vertical wavefront curvature at distance 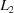 behind the nanofocusing optic
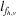	m	§2.4	Horizontal and vertical r.m.s. lateral coherence length at distance 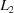 behind the nanofocusing optic
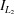	[intensity]	Equation (29)	Maximal intensity at distance 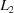 behind the nanofocusing optic
*T*	1	§2.4	Transmission of nanoprobe
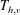	1	Equation (28)	Horizontal and vertical factor of transmission
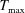	1	§4	Transmission for optimal diffraction-limited focusing
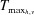	1	Equation (32)	Horizontal and vertical factor of transmission for optimal diffraction-limited focusing

Nanobeam properties			
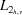	m	Equation (21)	Horizontal and vertical position of nanofocus
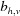	m	Equation (22)	Horizontal and vertical FWHM beam size in nanofocus
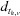	m	Equation (23)	Horizontal and vertical FWHM size of Airy disc
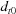	m	§2.4	FWHM size of Airy disc of homogeneously illuminated refractive lens
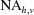	1	Equation (23)	Horizontal and vertical effective numerical aperture of the nanoprobe
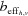	m	§2.4	Horizontal and vertical FWHM effective geometric beam size
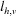	m	Equation (24)	Horizontal and vertical FWHM coherence length in nanofocus

**Table 2:** Optimal fraction of undulator radiation focused to the diffraction limit (λ = 1 Å) 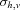
 are the r.m.s. electron beam sizes in the horizontal and vertical direction, respectively, and *T* is the fraction of the undulator radiation transmitted through the nanofocusing optic. 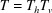 is separable into a horizontal (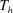) and vertical (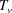) contribution [*cf.* equation (28)]. The parameters are taken from (Sette, 2012 ▶) and http://photon-science.desy.de/facilities/petra_iii/machine/parameters/index_eng.html.

		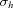 (µm)	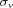 (µm)	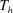	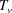	*T*
ESRF	High-β	395	3.9	0.0017	0.29	0.05%
	Low-β	50	3.9	0.002	0.29	0.06%
ESRF	Upgrade II	23.5	3.7	0.035	0.30	1.05%
PETRA III	High-β	141	4.9	0.0057	0.22	0.13%
	Low-β	34.6	6.3	0.0077	0.18	0.14%
DLSR	10 pm rad round	1.3	1.3	0.49	0.49	24%
